# Impacts of Omaha System-Based Continuing Care on the Medication Compliance, Quality of Life, and Prognosis of Coronary Heart Disease Patients After PCI

**DOI:** 10.21470/1678-9741-2021-0222

**Published:** 2022

**Authors:** Sijuan Yin, Yangyi Ou, E Ting

**Affiliations:** 1 Department of Cardiology, The Central Hospital of Wuhan, Tongji Medical College, Huazhong University of Science and Technology, Wuhan, Hubei Province, People’s Republic of China

**Keywords:** Percutaneous Coronary Intervention, Medication Adherence, Continuing Care, Coronary Heart Disease, Personal Satisfaction, Quality of Life.

## Abstract

**Introduction:**

The objective of this study is to explore the impacts of Omaha System-based continuing care on medication compliance, quality of life (QOL), and prognosis of coronary heart disease (CHD) patients after percutaneous coronary intervention (PCI).

**Methods:**

A total of 100 CHD patients who were hospitalized and received PCI were selected and divided into the control group and the observation group, 50 patients per group, according to a random number table method. The control group was given routine care, while the observation group was applied Omaha System-based continuing care on the basis of the control group.

**Results:**

Follow-up demonstrated that the Morisky-Green score of the observation group was significantly higher than that of the control group (P<0.001), indicating that the medication compliance of the observation group was significantly better than that of the control group (P<0.001). The short form-36 (SF-36) scores were notably higher after nursing compared with on admission; SF-36 scores of the observation group were significantly increased than those of the control group (P<0.001). The incidence of major adverse cardiac event (MACE) in the observation group was significantly lower than in the control group (P<0.001). The nursing satisfaction of the observation group was considerably higher than that of the control group (P<0.01).

**Conclusion:**

Omaha System-based continuing care could improve the medication compliance and QOL, reduce the incidence of MACE, and benefit the prognosis of CHD patients after PCI.

**Table:** 

Abbreviations, Acronyms & Symbols	
BMI	= Body mass index
CHD	= Coronary heart disease
ESC	= European Society of Cardiology
MACE	= Major adverse cardiac event
PCI	= Percutaneous coronary intervention
QOL	= Quality of life
SF-36	= Short form-36

## INTRODUCTION

Coronary heart disease (CHD) refers to a type of heart disease featured by coronary atherosclerosis, coronary luminal stenosis, and blockage leading to ischemia, hypoxia, and even necrosis of the functional myocardium that is responsible for the blood circulation. Currently, the incidence of CHD is increasing year by year globally, which is one of the major causes of death^[1]^. In clinical practice, percutaneous coronary intervention (PCI) that is based on drug therapy is an effective approach for the treatment of CHD. PCI can directly unblock the vascular stenosis caused by atherosclerosis, increase coronary blood flow, and effectively alleviate myocardial ischemia, which exerts critical effects on improving the clinical symptoms and prognosis of CHD patients^[2]^.

Although PCI can effectively treat CHD, benefit the quality of life (QOL) of patients, and improve long-term prognosis, relevant precautions and problems after PCI are often neglected by patients. The European Society of Cardiology (ESC) guidelines recommend that patients after PCI should take dual anti-platelet drugs at least for 12 months to minimize the incidence of major adverse cardiac event (MACE), including recurrence of angina, myocardial infarction, in-stent restenosis, and sudden cardiac death^[2]^. Studies have shown that CHD patients who have achieved good outcomes via PCI treatment during the hospitalization may still experience recurrence of the disease after hospital discharge and it results in a decline of QOL. The main reason is that discharged CHD patients fail to follow doctor’s advice to take the medication due to contempt, financial concern, etc., which greatly increases the recurrence rate of MACE after PCI, compromises the QOL of CHD patients after PCI, and escalates the risk of MACE and mortality^[3]^. How to conduct effective nursing interventions for rehabilitated patients, improve their medication compliance, reduce the incidence of MACE, and improve their QOL have become a major issue that needs to be paid attention to and resolved in clinical care. In recent years, the concept of continuing care has been proposed, which is considered to be an essential aspect of high-quality health service. It has been reported that continuing care can improve health outcomes and reduce the patients’ rehospitalization rate after acute hospitalization^[4]^. It is generally accepted that continuing care refers to a series of actions to ensure the coordination and continuity of the services when patients are translocated between different locations and different levels of health service in the same location. Additionally, keywords such as “coordination, connection, and consistency” are included and proposed by different scholars^[5]^.

Due to lacking a consistent definition regarding the concept and mode of continuing care, in recent years, it has been proposed the use of the Omaha System as a benchmark to regulate the specific aspects of continuing care. The Omaha System is a novel classification system of nursing practice developed by the American Omaha Visiting Nurse Association for community nursing, which has developed into a comprehensive classification system containing three sub-systems: problem classification scheme, intervention scheme, and problem rating scale for outcomes after more than 40 years of development^[6]^. The Omaha System is endorsed by the American Nurses Association (or ANA) as one of the supporting standards of nursing practice in the United States of America, which has been widely used in the field of nursing research and education^[7^,^8]^. Studies have revealed that the Omaha System-based continuing care can improve the behavior and QOL of CHD patients after PCI^[9]^. However, there is still a lack of relevant reports in terms of the impact of Omaha System-based continuing care on CHD patients after PCI. Based on existing studies, this paper aimed to comprehensively investigate the influence of Omaha System-based continuing care on medication compliance, QOL, and prognosis of CHD patients after PCI.

## METHODS

### General Information

This prospective study was approved by the Ethics Committee of The Central Hospital of Wuhan, Tongji Medical College, Huazhong University of Science and Technology (201-T). A total of 100 CHD patients who were hospitalized in our department and received PCI from January 2018 to March 2019 were selected. Inclusion criteria were: (1) being in accordance with relevant international diagnostic criteria for CHD by the 2017 ESC guidelines and diagnosis of CHD^[2]^; (2) the first PCI treatment done successfully without severe complications; (3) good cognitive function to complete the survey and follow-up; and (4) the patients informed and signed the consent. Exclusion criteria were: (1) participants with mental illness or cognitive dysfunction; (2) combined with important organ dysfunction, severe infection, malignancies, or other major diseases; (3) combined with congenital heart disease, valvular heart disease, cardiomyopathy, myocarditis, etc.; and (4) age < 18 or > 85 years old. The patients were divided into the observation group and the control group, 50 participants in each group, by a random number table method, in which the control group was received routine care and follow-up strategy, while in the observation group was applied the Omaha System-based continuing care on the basis of the control group.

### Data Collection

Patients’ data were recorded and collected, including age, gender, body mass index (BMI), whether or not combined with hypertension, diabetes, hyperlipidemia, whether or not smoking/drinking, physical conditions (respiration, pulse, blood pressure, heart rate), general clinical examinations (blood examination, coagulation, biochemistry), CHD type, PCI mode, short form-36 (SF-36) QOL score on admission and at nine months of follow-up after hospital discharge, Morisky-Green score at nine months of follow-up after discharge, MACE nine months after discharge, etc.

### Process

Both groups of CHD patients received standard drug treatment, including β-receptor antagonist, angiotensin-converting enzyme inhibitor, antiplatelet aggregation drug, lipid-lowering statin medication, etc.

According to the traditional nursing mode and existing researches, the control group was given routine care and follow-up after hospital discharge - Health education: patients were educated the basic knowledge of CHD, treatment methods, and daily relevant precautions^[9]^. Diet and exercise guidance: patients were recommended with low-salt and low-fat diets; patients with diabetes were provided with diet guidance for diabetes, prevented from uptake of high-oil, high-sugar, and hard, indigestible, and spicy food, while CHD patients were informed of the importance of eating less along with more meals. Psychology guidance and care: patients were received proactive communication to resolve their anxiety, worry, stress, agitation, and other negative emotions. Exercise guidance: patients were encouraged to take appropriate amount of exercises, avoiding excessively vigorous improper exercises or completely no exercises. Standardized medication guidance: patients were informed of the effects and precautions of relevant drugs, the importance of medication compliance, and possible adverse consequences of self-discontinuation of the medication. Follow-up after hospital discharge: patients were given the contact information when discharged to inform them of the precautions and the time and content during return visit.

On the basis of the control group, the observation group was conducted with the Omaha System-based continuing care according to existing research, which included as follows:

Establishment of the Omaha continuing care team: one chief physician of the cardiology department, who was responsible for the treatment plan, evaluation of intervention effects, and correction of guidance; seven nurses, including one head nurse of the cardiology department and two with a master degree of nursing, two nursing specialists, and two primary nurses - all team members had received all relevant trainings on the Omaha System and passed the examinations.

Evaluation and summary of problems: the health condition of CHD patients after PCI were evaluated via the Omaha System-based continuing care and effectiveness evaluation form. The purpose was to find existing and potential problems, analyze, and summarize these problems to propose corresponding intervention strategies.

Intervention based on the raised questions: according to the Omaha System, the intervention was divided into four categories, including: (1) strengthening health education: on the basis of general health education, PCI-related introductions, propaganda manuals, operating procedures, videos, etc. were provided to patients, their family, and the community health service center; (2) treatment and operation procedures: professional skills to reduce or prevent relevant symptoms were provided to patients after PCI, their family, and the community (such as informing patients of the observation of subcutaneous hemorrhage using antiplatelet drugs, observation and prevention of liver damage and myopathy by using statins, emergency treatment of recurring angina, etc.); (3) case management: in addition to traditional call return visits, it was encouraged to use advanced methods and resources (such as WeChat, QQ, and other social network tools) and adopt approaches such as coordination, encouragement, and cooperation with the community health service center to improve self-management awareness of patients after PCI and the management provided by their family and community, to answer questions online for patients by their family members and organizations, to encourage the communication of patients after PCI, and to set an example of self-management of patients in order to influence the entire group; (4) supervision: patients after PCI were supervised and tracked via various methods and interfered with the medication compliance. At the same time, intervention results were collected for the subsequent evaluation.

Criteria and evaluation of intervention effects: (1) medication compliance: Morisky-Green score was used as the evaluation criterion (four questions in total, including “Have you ever forgotten to take your medications?”, “Do you sometimes not take your medications?”, “You feel that your symptoms are under control, have you ever stopped taking medications?”, “When your symptoms worsened, have you ever stopped taking medications?”; answer “yes” to get 0 point, answer “no” to get 1 point, four points in total. A score of 4 indicated good medication compliance, while < 4 indicated poor medication compliance)^[10]^. (2) QOL: the QOL score (SF-36) was applied as the QOL evaluation standard (including overall health, vitality, mood, social function, and body function). The higher the score, the better the QOL; (3) healthy behavior: the Omaha System outcome evaluation system was used to evaluate patients’ diet management, medication management, exercise management, and so forth based on the cognition, behavior, and condition, with a full score of 5 for each item. The higher the score, the better the patients’ behavioral management; (4) patients’ MACEs (including unstable angina, myocardial infarction, heart failure, and sudden cardiac death) were recorded for nine months after hospital discharge by phone calls, WeChat, QQ, and clinical and rehospitalization medical records. Follow-up for nine months after the discharge, medication compliance, QOL, and MACE of the two groups of patients were compared.

### Outcome Measurements

Primary measurements: differences of medication compliance between the two groups of patients nine months after hospital discharge were observed and compared; changes and differences of QOL between the two groups of patients upon admission and nine months after the discharge were observed and compared; and MACE and prognosis between the two groups of patients nine months after the discharge were observed and compared.

Secondary measurements: follow-up for nine months after hospital discharge; the nursing satisfaction between the two groups were compared. The home-made nursing satisfaction survey questionnaire was used as a tool to record patients’ opinions to evaluate the nursing satisfaction, with a total of 100 points, which was grouped as very satisfied (80-100 points), generally satisfied (60-79 points), and unsatisfied (< 60 points) - nursing satisfaction = (very satisfied + general satisfaction)/total cases × 100%.

### Statistical Analysis

The IBM Corp. Released 2019, IBM SPSS Statistics for Windows, version 26.0, Armonk, NY: IBM Corp. software was used to process and analyze the data. The quantitative data were expressed as mean ± standard deviation ( $\overset{­}{x}$ ± SD). Independent *t*-test was applied for the comparison between groups, while paired *t*-test was conducted for the comparison before and after continuing care within the same group. The counting data were presented as case + percentage (n, %), and χ^^2^^ test was used for the analysis. If the expected counts > 20%, it indicated that the χ^^2^^ test was not feasible, so a Fisher’s exact test was conducted instead. *P*<0.05 indicated the statistically significant difference.

## RESULTS

### Comparison of General Information Between the Two Groups

There was no difference in terms of age, gender, BMI, smoking, drinking, hypertension, diabetes, hypercholesterolemia, CHD classification, New York Heart Association (or NYHA) functional classification on admission, and PCI between the observation group and the control group (all *P*>0.05; Table 1).

**Table 1:** Comparison of general information between the two groups.

Category	Observation group	Control group	χ^^2^^ test	*P*-value
Case (n)	50	50		
Age (years)	67.1±7.8	69.5±7.5	0.853	0.40
Gender (male)	24	26	0.185	0.67
BMI (kg/m^^2^^)	24.74±2.98	24.12±3.01	1.043	0.30
Smoking	38	40	0.218	0.64
Drinking	35	33	0.182	0.67
Hypertension	28	29	0.182	0.67
Diabetes	25	23	0.501	0.48
Hypercholesterolemia	29	27	0.481	0.49
Cerebrovascular diseases	28	30	0.741	0.39
Education level			0.745	0.86
College	10	10		
High school	22	20		
Junior middle school	12	15		
Primary school and below	6	5		
CHD classification			0.863	0.94
Unstable angina	19	20		
Stable angina	14	11		
Acute myocardial infarction	10	9		
Ischemic cardiomyopathy	6	8		
Others	1	2		
Cardiac function on admission			0.249	0.97
I	9	10		
II	16	16		
III	20	18		
IV	5	6		
PCI types			0.764	0.59
Percutaneous transluminal coronary angioplasty	14	11		
Coronary stent implantation	32	35		
Coronary thrombus aspiration	1	2		
Others	3	2		

BMI=body mass index; CHD=coronary heart disease; PCI=percutaneous coronary intervention

### Comparison of Medication Compliance Between the Two Groups After Continuing Care

**Fig. 1:**
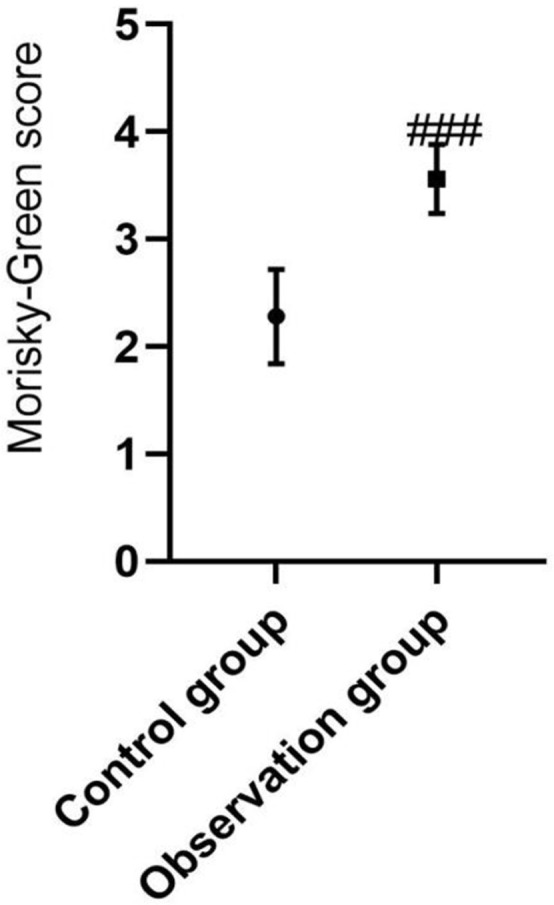
Comparison of Morisky-Green score between the two groups after continuing care. Compared with the control group after continuing care, ^###^P<0.001.

Nine-month follow-up after hospital discharge showed that the Morisky-Green score of the observation group was significantly higher than that of the control group (*P*<0.001), and the number of patients with good medication compliance in the observation group was significantly higher than in the control group (*P*<0.001; Table 2 and Figure 1).

**Table 2:** Comparison of medication compliance between the two groups after continuing care.

Group	Case (n)	Morisky-Green score	Medication compliance (n, %)
Observation group	50	3.56±0.32	46 (92%)
Control group	50	2.28±0.44	24 (48%)
χ^^2^^ test		16.642	19.238
*P*-value		< 0.001	< 0.001

### Comparison of the QOL Between the Two Groups Before and After Continuing Care

**Fig. 2:**
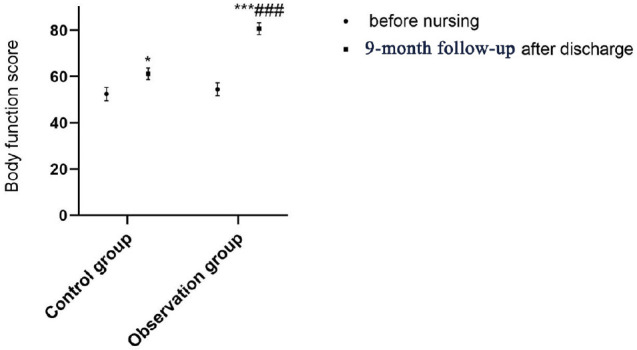
Comparison of body function score between two groups before and after continuing care. Compared with on admission within the same group, *P<0.05, ***P<0.001; compared with the control group after continuing care, ^###^P<0.001.

**Fig. 3:**
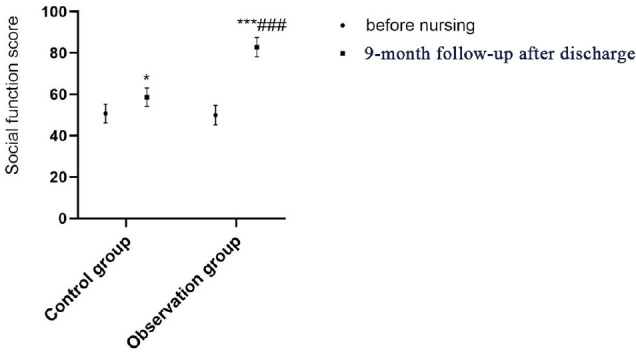
Comparison of social function between the two groups before and after continuing care. Compared with on admission within the same group, *P<0.05, ***P<0.001; compared with the control group after continuing care, ^###^P<0.001.

**Fig. 4:**
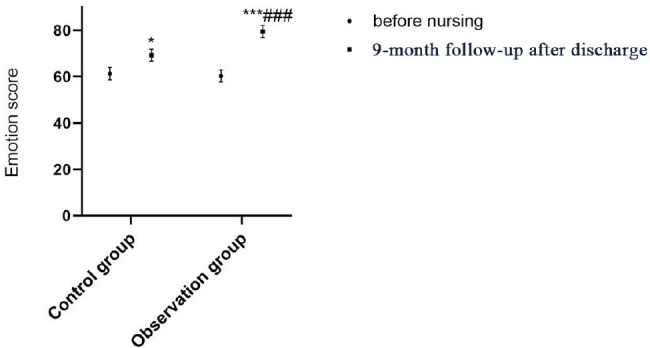
Comparison of emotional scores between the two groups before and after continuing care. Compared with on admission within the same group, *P<0.05, ***P<0.001; compared with the control group after continuing care, ^###^P<0.001.

**Fig. 5:**
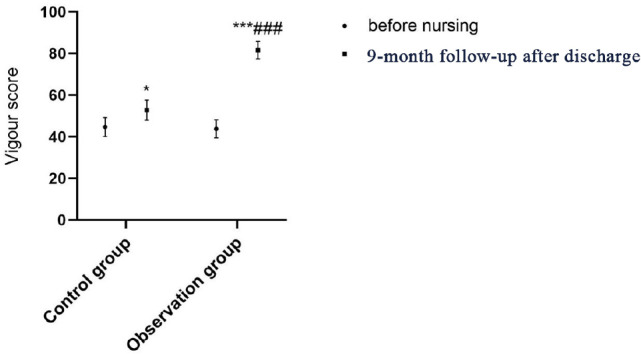
Comparison of vitality scores between the two groups before and after continuing care. Compared with on admission within the same group, *P<0.05, ***P<0.001; compared with the control group after continuing care, ^###^P<0.001.

**Fig. 6:**
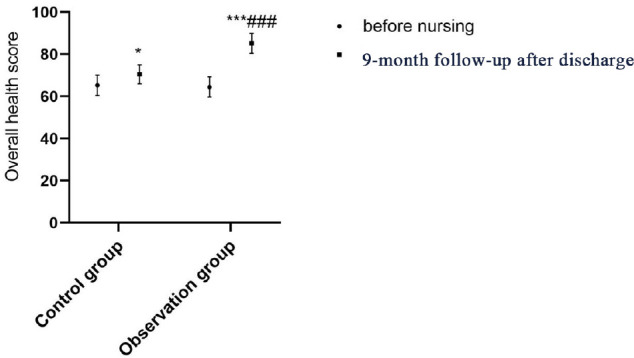
Comparison of overall health score between the two groups before and after continuing care. Compared with on admission within the same group, *P<0.05, ***P<0.001; compared with the control group after continuing care, ^###^P<0.001.

There was no difference regarding SF-36 scores between the two groups on admission (all *P*>0.05); after continuing care and post-discharge nine-month follow-up, the SF-36 scores of the two groups were both significantly increased (all *P*<0.05), of which the SF-36 score of the observation group was significantly higher than that of the control group (*P*<0.001; Table 3 and Figures 2-6).

**Table 3:** Comparison of QOL between the two groups before and after continuing care.

Group	Observation group (n=50)		Control group (n=50)	
**Time**	**On admission**	**9-month follow-up**	**On admission**	**9-month follow-up**
Physical function	52.44±2.84	61.24±2.48^*^	54.52±2.82	80.72±2.56^***###^
Social function	50.68±4.51	58.62±4.38^*^	49.94±4.71	82.79±4.63^***###^
Emotion	61.32±2.61	69.28±2.54^*^	60.29±2.57	79.49±2.63^***###^
Vitality	44.67±4.52	52.77±4.83^*^	43.76±4.34	81.62±4.23^***###^
Overall health	65.28±4.82	70.52±4.54^*^	64.47±4.78	85.19±4.73^***###^

Compared with on admission within the same group, ^*^P<0.05, ^***^P<0.001; compared with the control group after continuing care, ^###^*P*<0.001. QOL=quality of life

### Comparison of MACE Between the Two Groups After Continuing Care

In the nine-month follow-up after hospital discharge, the incidence of MACE in the observation group was significantly lower than in the control group (*P*<0.001; Table 4).

**Table 4:** Comparison of MACE between the two groups after continuing care.

Group	Observation group	Control group	*P*-value
Case (n)	50	50	
Unstable angina	6	16	0.03
Non-fatal myocardial infarction	1	5	< 0.001
In-stent restenosis	1	5	< 0.001
Sudden cardiac death	0	2	0.50
Total	8	28	< 0.001

MACE=major adverse cardiac event

### Comparison of Nursing Satisfaction Between the Two Groups After Continuing Care

In the nine-month follow-up after discharge, the nursing satisfaction of the observation group was higher than that of the control group (*P*<0.01; Table 5).

**Table 5:** Comparison of nursing satisfaction between the two groups after continuing care.

Group	Observation group	Control group	*P*-value
Case (n)	50	50	
Very satisfied	42	22	< 0.001
Generally satisfied	6	15	0.03
Unsatisfied	2	13	< 0.01
Overall satisfaction	48 (96%)	37 (74%)	< 0.01

## DISCUSSION

It is very common that MACE occurs on CHD patients after PCI due to poor medication compliance after hospital discharge, which greatly compromises patients’ QOL while increases the risk and death resulted from drug withdrawal^[11]^. Traditional hospital care and follow-up mode can no longer meet the requirements of long-term recovery for patients after PCI. Thus, continuing care has been widely applied in a variety of clinical practices, which has been shown to improve patients’ medication compliance and their QOL^[12^-^15]^. Studies have also revealed that continuing care can improve the medication compliance and QOL of patients after PCI^[16]^. In this study, the medication compliance of the observation group is significantly higher than that of the control group after nine months of follow-up after hospital discharge, suggesting that Omaha System-based continuing care can improve medication compliance of CHD patients after PCI, which is consistent with previous studies^[17]^. The reason could be due to the fact that Omaha System-based continuing care can strengthen the health knowledge of patients on one hand, and on the other hand, it could educate patients to be aware of relevant diseases, the importance of taking medications, and the risk of discontinuation of medications. The dynamic collection and evaluation of patients’ recovery after PCI and the implementation of relevant interventions have motivated the enthusiasm of patients, their family, and communities, ultimately leading to the improvement of patients’ medication compliance.

QOL, also known as quality of living and quality of survival, refers to the degree to which people think about their own goals, standards, expectations, and the state of life they value. With the ongoing progress and development of the nursing mode, the ultimate goal of nursing is no longer limited to simply extending the survival of patients whereas focusing more to improve the QOL of patients. Currently, studies have demonstrated that the Omaha System-based continuing care can improve the QOL of patients with cervical cancer and chronic obstructive pulmonary disease^[18^,^19]^. In this study, there is no difference regarding the QOL scores between the two groups of patients upon admission. After continuing care and post-discharge nine-month follow-up, QOL scores of the two groups of patients are significantly increased, and QOL scores of the observation group are significantly higher than those of the control group, suggesting that the Omaha System-based continuing care can better improve the QOL of CHD patients after PCI. These data are also consistent with the results in existing studies, which is thought to be mainly due to the fact that Omaha System-based continuing care can improve patients’ medication compliance that has been shown to improve the QOL of patients with CHD and myocardial infarction^[20^,^21]^. In addition, studies have revealed that continuing care, psychological care, and other relevant approaches could improve the QOL of CHD patients after PCI by enhancing their psychological state and social behavior^[22]^.

Poor medication compliance of CHD patients after PCI can result in various MACEs. Improving patient medication compliance can help to reduce the incidence of MACE^[23]^. Our study has shown that after continuing care and post-discharge nine-month follow-up, the medication compliance is notably increased, whereas the incidence of MACE is considerably lower in the observation group in comparison to the control group, which is in line with the existing data. There are no sudden cardiac death events in the observation group, indicating that the Omaha System-based care can reduce the incidence of MACE in CHD patients after PCI and improve their prognosis.

Nursing satisfaction utilizes the investigation and follow-up to understand patients’ satisfaction degree with the clinical nursing, which is helpful to check caveats and fill gaps, improve and optimize nursing, continue to strengthen service philosophy, and ultimately improve the quality of clinical nursing service. In this study, after continuing care and post-discharge nine-month follow-up, the nursing satisfaction of the observation group is significantly higher than that of the control group, suggesting that Omaha System-based continuing care can improve the nursing satisfaction of patients after PCI.

### Limitations

There are still some limitations and shortcomings in this study, for example, the overall sample size is small, multi-center studies for simultaneous comparison need to be conducted, more comprehensive observations and studies are missing (a study has reported that the Omaha System-based continuing care can improve the QOL of patients through psychological intervention), the observation and follow-up period are short, etc.^[22]^. In the future, it is worthwhile to expand the sample size, extend the follow-up time, and leverage a multi-center study for further in-depth research. How to institutionalize and simplify the nursing intervention in order to transform it into routine part of clinical nursing warrants the future investigation.

## CONCLUSION

In summary, Omaha System-based continuing care can improve the medication compliance of CHD patients after PCI, enhance their QOL, reduce the incidence of MACE, improve patients’ prognosis, and increase nursing satisfaction, which is worthy of clinical applications.

**Table:** 

Authors’ Roles & Responsibilities	
SY	Substantial contributions to the concept and design of the work; revising the work critically for important intellectual content; agreement to be accountable for all aspects of the work in ensuring that questions related to the accuracy or integrity of any part of the work are appropriately investigated and resolved; final approval of the version to be published
YO	Substantial contributions to the acquisition, analysis and interpretation of data for the work; final approval of the version to be published
TE	Substantial contributions to the analysis of data for the work; drafting the work and revising it critically; final approval of the version to be published
